# Marital dissolution and cognition: The mediating effect of Aβ neuropathology

**DOI:** 10.1002/dad2.70032

**Published:** 2024-10-29

**Authors:** Avinash Chandra, Rifah Anjum, Sheena Waters, Petroula Proitsi, Laura J. Smith, Charles R. Marshall, for the Alzheimer's Disease Neuroimaging Initiative

**Affiliations:** ^1^ Centre for Preventive Neurology, Wolfson Institute of Population Health Queen Mary University of London London UK

**Keywords:** Alzheimer's disease, amyloid beta, divorce, episodic memory, stress, widowhood

## Abstract

**INTRODUCTION:**

Widowhood and divorce are extremely stressful life events that are associated with dementia, but the neurobiological underpinnings of this risk remain unknown. Amyloid beta (Aβ) load may explain influences of chronic stress, commonly seen in disruptive marital transitions, on cognitive decline.

**METHODS:**

We examined whether Aβ quantified by tracer uptake on positron emission tomography mediates associations between marital dissolution and executive functioning and episodic memory performance using data from 543 cognitively normal (CN) participants from the Alzheimer's Disease Neuroimaging Initiative.

**RESULTS:**

Marriage dissolution was associated with increased Aβ burden (*β* = 0.56; *P *= 0.015) and worse memory performance (*β* = –0.09; *P *= 0.003). Aβ levels were a significant mediator for the relationship between marriage dissolution and memory (average causal mediation effect = –0.007; *P *= 0.029).

**DISCUSSION:**

Findings suggest that stressful life events, such as the dissolution of one's marriage, might exert an effect on Alzheimer's disease proteinopathy, which may subsequently influence poor cognition.

**Highlights**

- Marital dissolution was associated with increased amyloid beta (Aβ) and memory declines.
- Aβ burden mediated associations between marital dissolution and memory.
- Findings were robust to potential non‐linear influences of age.
- Mediation results were not observed when stratifying marital groups by sex.

## 1 INTRODUCTION

Major stress may adversely influence brain health and heighten the risk of dementia.^1^ Dissolution of marriage, through either divorce or death of a spouse,^2^ is common and has been identified as among the most stressful events across the adult lifespan.^3^ These experiences may result in poorer mental health and declines in health‐related quality of life.^4^, ^5^ Moreover, marital dissolution is associated with an increased risk of cognitive impairment and dementia.^6^, ^7^, ^8^ The reasons for this are likely multifactorial and include limited access to shared economic and sociopsychological resources, encompassing social engagement and support, which promote better cognitive health after marital transitions.^6^ Adverse neurobiological impacts may also stem indirectly from the stress associated with marital disruption. For example, persistent stress may promote unhealthy behaviors and maladaptive coping such as excessive smoking and drinking, which can lead to central nervous system (CNS) damage.^6^, ^9^

Stressful life events, such as marital dissolution, may have more direct effects on elements comprising the neuropathological cascade of Alzheimer's disease (AD).^10^ This includes toxic effects of chronic stress on hippocampal neurons,^11^ neuroinflammation and focal neurodegeneration,^12^ and pathogenic protein accumulation, including amyloid beta (Aβ) aggregation.^13^ Aβ is thought to be a driving force facilitating this cascade.^10^ While no direct relationship has yet been established between marital dissolution and Aβ pathology,^14^, ^15^, ^16^ previous research has found an interaction between amyloid and widowhood on cognitive decline. Widowed individuals who exhibited high levels of brain Aβ had steeper trajectories of cognitive decline.^15^ However, no study has yet examined the mediating influences of cerebral Aβ levels on the relationship between both divorce and widowhood and domain‐specific cognitive performance. Thus, the primary aim of the current study was to evaluate whether Aβ pathology measured through in vivo neuroimaging mediates influences of marriage dissolution on executive functioning (EF) and episodic memory (EM) performance in healthy older adults.

Exploring these effects in a healthy population could shed further light on the pathogenic mechanisms that underlie the role of psychosocial stress in contributing to the earliest signs of AD. These individuals are at a stage, well before the onset of any clinically meaningful symptomatology, when the benefits of interventions and prevention strategies (e.g., social support, counseling, and access to community resources) may be at their most efficacious.

## 2 METHODS

### 2.1 Participants and data source

**FIGURE 1:**
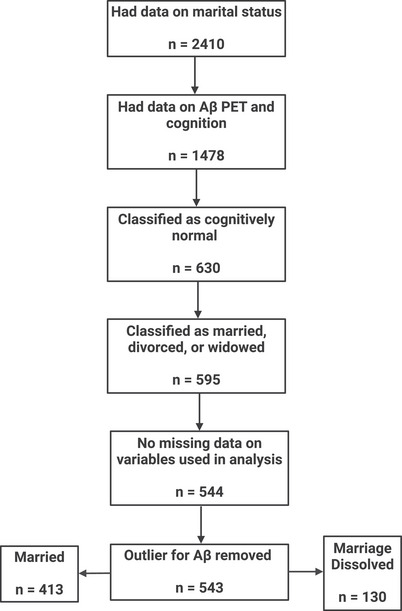
Sample size flowchart for ADNI subjects used in the current analysis. Created with BioRender.com. Aβ, amyloid beta; ADNI, Alzheimer's Disease Neuroimaging Initiative; PET, positron emission tomography

Data used in this study were obtained from the Alzheimer's Disease Neuroimaging Initiative (ADNI; available at adni.loni.usc.edu). ADNI is a multicenter and longitudinal study that prospectively recruits participants who are either cognitively normal (CN) or have diagnoses of mild cognitive impairment (MCI) or AD. The primary aim of ADNI is the early identification of features associated with AD, in addition to assessing its progression, through the integration of neuroimaging measures and other biomarkers with cognitive and clinical information. The current investigation used cross‐sectional data from 543 CN subjects from ADNI (Figure 1), who were either married or had their marriage dissolved (through divorce or death of spouse). Data were also available on age and cognitive performance at Aβ positron emission tomography (PET) scan visit, sex, education level, and apolipoprotein E (*APOE*) ε4 genotype.

**RESEARCH IN CONTEXT**

1. **Systematic review**: Using traditional sources, the authors reviewed the literature on psychosocial dementia risk factors and associated neurobiological features. While some evidence shows that amyloid beta (Aβ) may play a role in cognitive decline associated with stressful life events, no study has explored whether Aβ can directly mediate influences of marital dissolution on domain‐specific cognitive decline.
2. **Interpretation**: Findings suggest a potential neurobiological pathway, consisting of increased Aβ burden, through which marital dissolution, either through divorce or widowhood, might impact declines in memory.
3. **Future directions**: This article highlights the importance of marital dissolution as a potential risk factor for early Alzheimer's disease symptomatology and how this may occur mechanistically. Findings could help to inform interventions for dementia prevention and to identify specific groups warranting priority for anti‐amyloid treatment. Future research should aim to validate these findings using more well‐powered and diverse samples, in addition to longitudinal approaches.

### 2.2 Measures

#### 2.2.1 Marital status

Study participants reported their marital status at the time of the ADNI baseline visit. There were five response categories including: married, divorced, never married, unknown, and widowed. For current analyses, participants were categorized into one of two marital groups. The first were those who reported being married at baseline (control group), while the second were those who reported either being either widowed or divorced (“marriage dissolved” group).

#### 2.2.2 Aβ PET imaging

Amyloid PET neuroimaging acquisition and preprocessing were undertaken within the ADNI study using either the radiotracers [^18^F]florbetapir (Amyvid) or [^18^F]florbetaben (Neuraceq). For [^18^F]florbetapir: participants were injected with a 370 ± 37 MBq bolus, and 3D amyloid PET scans were acquired 50 to 70 minutes postinjection, which lasted ≈ 20 minutes and consisted of four X 5 minute frames. For [^18^F]florbetaben: participants were injected with a 300 ± 30 MBq bolus, and 3D amyloid PET scans were acquired 90 to 110 minutes postinjection, which lasted ≈ 20 minutes and consisted of four X 5 minute frames. Preprocessing steps included magnetic resonance imaging (MRI) segmentation and parcellation using FreeSurfer 7.1.1, co‐registration between respective MRI and PET images, intensity normalization, and the calculation of standardized uptake value ratios (SUVR) including cortical summary SUVR values for both florbetapir and florbetaben. Further information on amyloid PET processing methods can be found on the ADNI website and previous work.^17^ Only scans that fully passed quality control and had no evidence of technical errors were selected for the current investigation.

Data from [^18^F]florbetapir and [^18^F]florbetaben were also used to calculate standardized Centiloid transformations by ADNI investigators, which allows for the direct comparison and harmonization of Aβ levels derived from both radiotracers.^18^, ^19^ Briefly, direct normalized cortical SUVR to Centiloid unit conversion was conducted by adapting the level‐2 analysis developed by Klunk et al.^20^ Techniques used in this conversion included linear regressions, scaling, and transformation equations.

#### 2.2.3 Cognition

Data on cognitive performance for the initial Aβ PET visit were quantified using a battery of standardized neuropsychological assessments. Cognitive tests used in the generation of the EF score included (1) category fluency–animals, (2) category fluency– vegetables, (3) Trail Making Test A, (4) Trail Making Test B, (5) digit span backward, (6) Wechsler Adult Intelligence Scale‐Revised (WAIS‐R) Digit Symbol Substitution, and items from the (7) Clock Drawing Test. Tests used for EM score generation included (1) Rey Auditory Verbal Learning Test (RAVLT), (2) Alzheimer's Disease Assessment Scale–Cognitive Subscale (ADAS‐Cog), (3) Wechsler Memory Scale‐Revised (WMS‐R) Logical Memory, and (4) recall tests from the Mini‐Mental State Examination (MMSE).

Composite EF and EM scores were derived using item response theory methodology and had a mean of 0 and a standard deviation (SD) of 1. A more in‐depth explanation of the processes and methods used to generate these composite scores can be found elsewhere.^21^, ^22^ These composite scores were chosen as they measure two neuropsychological domains that are typically impaired very early during mild AD, relative to other areas of cognition (e.g., language, sustained attention).^23^

### 2.3 Statistical analyses

The Shapiro–Wilk test and corresponding histograms were used to identify primary outcome variables with non‐normal distributions. All three (Aβ Centiloid value, EF score, and EM score) met this criteria (Table S1, Figures S1–S3 in supporting information) and, due to the inclusion of negative values, were cube root transformed to correct for this.^24^ Before the transformation, outliers were detected using the “qqplot” function in R^25^ with one outlier being identified for Aβ Centiloid score and removed from analyses (Figure S4 in supporting information). Using absolute *Z* value computation, this case was additionally categorized as an extreme outlier with a *Z* value > 5. In Aβ PET data using Centiloid values, quantification methods have been previously shown to influence the presence of outliers.^26^ Differences in demographic and baseline characteristics of the study sample split by marital status were conducted using independent samples *t* tests for continuous variables and chi‐square tests for categorical variables.

Multiple linear regression was first run to assess the relationship between (1) marriage dissolution and Aβ levels and (2) marriage dissolution and cognition. Covariates for regressions with Aβ as the outcome included age, sex, education level, PET tracer, and *APOE* ε4 status, while those for regressions with cognition (EF or EM score) as the outcome included age, sex, and education level. For results that demonstrated significant associations, interactions between age, sex, education, and/or *APOE* ε4 and marital dissolution were individually evaluated in separate secondary models (depending on the covariates used in initial models). This was done to examine whether associations between exposures and outcomes were different based on population characteristics. As part of this analysis, interactions were also tested between Aβ levels and marital dissolution to investigate moderating or synergistic effects on cognition. Regression models with significant associations were selected for two‐stage mediation analysis to determine whether Aβ pathology could mediate the potential influences of marriage dissolution on cognition.

The Baron and Kenney criteria^27^ were initially used to determine whether sufficient evidence was present for a mediation effect. Specifically, requirements were (1) *X* (marriage dissolution) is correlated with *Y* (cognition), (2) *X* (marriage dissolution) is correlated with *M* (Aβ levels), (3) *M* (Aβ levels) is correlated with *Y* (cognition)—while controlling for *X*, and (4) the correlation between *X* (marriage dissolution) and *Y* (cognition) is reduced when including M (Aβ levels) in the model (partial mediation) or disappears (total mediation). The second stage of mediation analysis examined causal mediation effects using the “mediation” R package.^28^ Specifically, we computed the average direct effect (ADE) and average causal mediation effect (ACME). These metrics respectively aim to quantify both direct and indirect mediated effects of Aβ levels on cognition. Non‐parametric bootstrapping consisting of 5000 simulations was used to generate bias‐corrected confidence intervals for ADE and ACME. Mediation analyses consisted of multiple regressions used in earlier analytic steps. Sensitivity analyses included re‐testing for mediation and moderation effects (1) within stratified groups for covariates that demonstrated a significant interaction in secondary models, (2) when including both age and age squared in original regression models (fully adjusted model to determine whether results differ when accounting for non‐linear effects of age),^29^ and (3) separately for the widowed and divorced groups. *P *< 0.05 was set as the threshold for statistical significance across all models. All statistical analyses were performed using R software version 4.2.2 (R Project for Statistical Computing). This research used Queen Mary's Apocrita HPC facility, supported by QMUL Research‐IT. https://doi.org/10.5281/zenodo.438045, in addition to Open OnDemand.^30^

## 3 RESULTS

### 3.1 Demographic results

Sample characteristics can be found in Table 1. Groupwise comparison indicated that there were more women in the marriage dissolved category relative to men. Moreover, participants in this group also had higher levels of Aβ pathology relative to those who were married. Otherwise, no significant group differences were observed.

**TABLE 1:** Sample characteristics.

Variable	Married (*n* = 413)	Marriage dissolved (*n* = 130)	*P* value
Age at Aβ PET scan (years), M (SD)	72.5 (6.6)	73.8 (7.7)	0.091
Education (years), M (SD)	16.7 (2.4)	16.2 (2.6)	0.068
Sex, no. (%male)	199 (48.2)	33 (25.4)	< 0.001^**^
Race, no. (%White)	376 (91)	113 (86.9)	0.230
Time between baseline visit and Aβ PET scan (months), M (SD)	8.4 (23)	4.9 (15.7)	0.052
*APOE* ε4 status, no. (%carrier)^a^	131 (31.7)	34 (26.2)	0.274
Aβ Centiloid level, M (SD)	16 (31.7)	24.2 (37.2)	0.023^*^
Aβ PET tracer, no. (%[^18^F]florbetapir)	310 (75.1)	96 (73.8)	0.871
EF composite score, M (SD)	0.8 (0.7)	0.6 (0.7)	0.066
EM composite score, M (SD)	1.1 (0.6)	1.0 (0.6)	0.129
Marriage dissolution, no. (%widowed)^b^	–	62 (47.7)	–

*Note*: This table presents demographic information and information on outcomes stratified by marital status.

Abbreviations: Aβ, amyloid beta; *APOE*, apolipoprotein E; EF, executive functioning; EM, episodic memory; M, mean; no., number; PET, positron emission tomography; SD, standard deviation.

^a^ In the married group 121 heterozygous and 10 homozygous carriers; in the marriage dissolved group 32 heterozygous carriers and 2 homozygous carriers.

^b^ Only applicable to marriage dissolution group. Pairwise comparison not possible. Values are relative to the divorced group.

* Significant at *P *< 0.05.

** Significant at *P *< 0.001.

### 3.2 Relationships among marriage dissolution, Aβ pathology, and cognition

**FIGURE 2:**
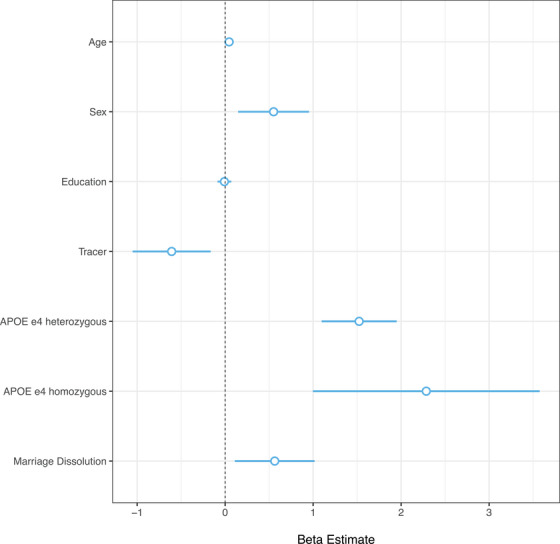
Associations between Aβ pathology and marriage dissolution. The forest plot displays the magnitude of regression coefficient estimates (*β*) for the model examining associations between marriage dissolution and Aβ neuropathology. Estimates on the *x* axis represent β coefficients and error bars represent the 95% confidence intervals. Aβ, amyloid beta; APOE, apolipoprotein E

**FIGURE 3:**
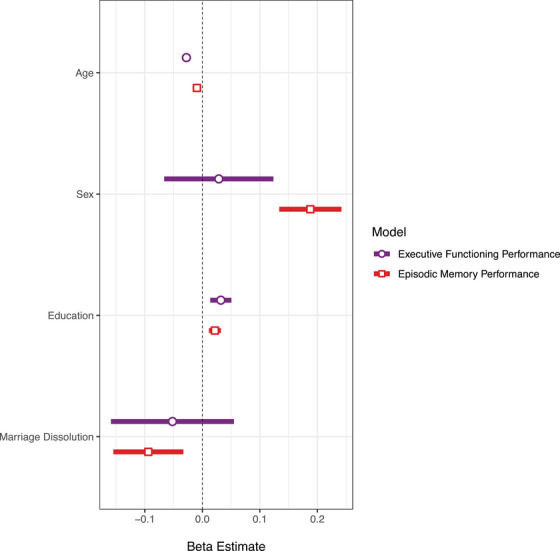
Associations between executive functioning and episodic memory performance and marriage dissolution. The forest plot displays the magnitude of regression coefficient estimates (*β*) for the two models examining associations between marriage dissolution and cognition. Estimates on the *x* axis represent *β* coefficients and error bars represent the 95% confidence intervals

**FIGURE 4:**
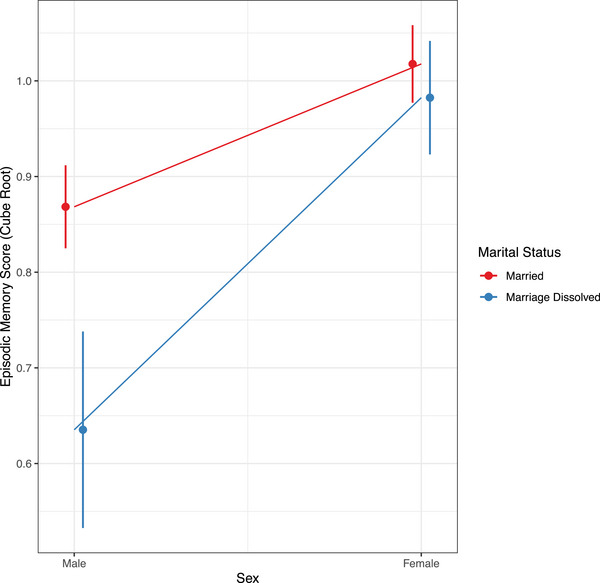
Interaction effect between marital dissolution and sex. The graphical plot indicates the magnitude and direction of the effect of the significant interaction term (marital status x sex) from the model. Error bars represent the 95% confidence intervals

Marriage dissolution was associated with increased levels of Aβ neuropathology quantified by standardized Centiloid values (*β* = 0.56; 95% confidence interval [CI]: 0.11 to 1.02; *P *= 0.015; Figure 2). An association was also observed between marriage dissolution and lower EM scores (*β* = −0.09; 95% CI: −0.15 to −0.03; *P *= 0.003; Figure 3) but not between marriage dissolution and EF scores (*β* = −0.05; 95% CI: −0.16 to 0.05; *P *= 0.340; Figure 3). Through secondary models, an interaction effect between marriage dissolution and sex on higher EM score was observed (*β* = 0.20; 95% CI: 0.07 to 0.33; *P *= 0.003; Figure 4). This indicated that the effect of marital dissolution on worse EM is greater in men than in women. No other significant interaction effects were found, including between Aβ and marital dissolution (Table S2 in supporting information).

### 3.3 Mediation analyses

#### 3.3.1 Baron and Kenney criteria for partial mediation

Given significant associations found among marriage dissolution, Aβ pathology, and EM scores, mediating influences of Aβ on the relationship between marriage dissolution and memory were evaluated. As indicated previously, criteria 1 and 2 were met. Specifically, significant associations were observed between (1) marriage dissolution and memory performance and (2) marriage dissolution and Aβ pathology. Aβ levels were negatively associated (when including marital dissolution in the model) with memory scores (*β* = −0.01; 95% CI: −0.02 to −0.002; *P *= 0.021). After including Aβ pathology in the regression equation, the relationship between marriage dissolution and EM scores becomes weaker in terms of effect size and significance value (Table 2). Thus, criteria 3 and 4 of the Baron and Kenney measures were met and sufficient evidence existed for a partial mediating effect of Aβ on the negative influence of marriage dissolution on memory performance.

**TABLE 2:** Mediation results using Baron and Kenney criteria.

Regression model	*β* ^a^	95% confidence interval^a^	*t* value	*P* value
(1) EM score ∼ Marriage dissolution	−0.094	−0.155 to –0.033	−3.03	0.003^*^
(2) EM score ∼ Marriage dissolution + Aβ	−0.088	−0.149 to –0.027	−2.83	0.005^*^

*Note*: This table presents partial mediation results with and without Aβ as a covariate.

Abbreviations: Aβ, amyloid beta; EM, episodic memory.

^a^ Rounded to three decimal places to demonstrate differences in effect size.

* Significant at *P *< 0.01.

#### 3.3.2 Causal mediation effects

**FIGURE 5:**
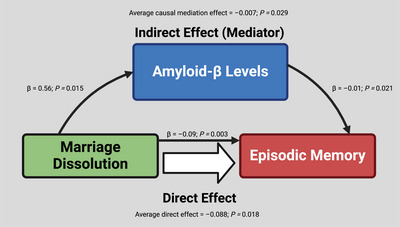
Diagram of mediation effects. The graphic indicates significant results from mediation analyses using the Barron and Kenney criteria and from causal mediation using non‐parametric bootstrapping. Estimates, *P* values, and corresponding lines are representative of individual regression models. Created with BioRender.com

Results from non‐parametric bootstrapping indicated that both direct and Aβ‐mediated effects for the association between marriage dissolution and memory performance were present. Estimates for the ADE (P*p *= 0.018) indicated a direct effect of marriage dissolution on memory scores when accounting for the mediation effect of Aβ, while those for the ACME indicated a mediation or indirect (*P *= 0.029) effect of Aβ for this relationship (Table 3; Figure 5).

**TABLE 3:** Mediation results using bootstrapping.

Outcome	Estimate^a^	95% confidence interval^a^	*P* value
ACME	−0.007	−0.017 to –0.0004	0.029^*^
ADE	−0.088	−0.167 to –0.016	0.018^*^
Total effect	−0.095	−0.173 to –0.024	0.009^**^
Proportion mediated	0.075	0.002 to 0.356	0.038^*^

*Note*: This table presents results from mediation results using non‐parametric bootstrap confidence intervals with the percentile method.

Abbreviations: ACME, average causal mediation effect; ADE, average direct effect; EM, episodic memory.

^a^ Rounded to 3 decimal places to highlight precise effect size.

* Significant at *P *< 0.05.

** Significant at *P *< 0.01.

### 3.4 Sensitivity analyses

When stratifying the sample by sex, no significant interaction or mediation effects were found. In fully adjusted models (accounting for age squared), mediation effects were still observable (Tables S3–S4 in supporting information). When examining only widowed participants, no significant mediation or interaction effects were observed. No mediation effects were found separately for divorced participants. There were no separate associations between widowhood or divorce and EF performance. Full results for all sensitivity analyses can be found in the supporting information.

## 4 DISCUSSION

Here we have shown that marital dissolution is associated with poorer EM function, and this association is mediated by Aβ burden. We evaluated relationships between marriage dissolution through either divorce or death of a spouse, Aβ levels quantified by Centiloid values, and cognitive performance on EF or EM, using a two‐stage analysis to determine a mediation effect for Aβ burden. Mediation results were robust to potential non‐linear influences of age and were not observed when stratifying marital groups by sex or separately examining widowhood and divorce, potentially due to loss of power. Overall, these findings further validate the importance of divorce and widowhood as risk factors for the development of cognitive impairment but go further than previous studies by demonstrating that this is partially mediated by influences of neuropathological change characteristic of AD, namely elevated CNS Aβ neuropathology.

Our findings complement previous research that identified in vivo Aβ pathology as a moderator of widowhood and cognitive decline. Prior work from Biddle et al. suggested that the experience of spousal death could interact or exert parallel influences with Aβ burden to exacerbate cognitive deterioration.^15^ However, this result was not observed in the current investigation using data from ADNI. Overall, sensitivity analysis did not indicate a substantially different effect for widowed participants separately compared to the combined marital dissolution group. While we adopted a cross‐sectional approach, the present study provides the first justification that Aβ burden may mediate the relationship between the stress affiliated with both the loss of a spouse through death or divorce and poorer performance on EM, which is one of the earliest clinical markers before the onset of AD.^31^ High levels of uncontrollable chronic stress, potentially attributable to factors like low levels of life satisfaction and unhappiness experienced after marital dissolution, can have more immediate neurobiological effects that may explain the current results.^13^

Preclinical research suggests that stress‐induced alterations in brain immune response can result in microglial dysfunction, which may in turn lead to an aggregation of CNS Aβ pathology through impairments in degradation and clearance.^32^ Furthermore, it was evidenced in non‐human primates that continuous glucocorticoid administration, typically released during stress, facilitated increases in Aβ_42_ plasma levels and reductions in insulin‐degrading enzyme mRNA.^33^ Exposure to long‐term stress may exacerbate Aβ accumulation, with corticosterone possibly intermediating these effects.^34^ Interestingly, we found no significant effects with respect to EF performance. EF has been linked to brain areas belonging executive control network (ECN), whereas EM has been linked to those that comprise the default mode network (DMN).^35^ Research has shown that even in MCI, ECN connectivity is not disrupted.^36^ However, even in cognitively healthy individuals, disruptions in the DMN, linked to memory decline, may be attributable, at least in part, to early Aβ neuropathology.^37^ This may be due to the neurotoxic effects of Aβ on metabolic processes within afflicted neurons.^38^ Therefore, within the current study, influences of chronic stress linked to marital dissolution on Aβ may have in turn impacted memory performance through DMN alterations in CN subjects. This neurobiological pathway may not be applicable to EF performance, which in this cognitively healthy sample may have been more reflective of mechanisms tied to cognitive reserve.

To our knowledge, this study provides the first evidence of a neurobiological mediator between marital dissolution (through either widowhood or divorce) and cognitive performance. This adds to other possible mediators of this relationship identified in the literature including mental and physical health,^39^ number of children,^40^ social integration, and financial resources.^41^ This study also highlights the importance of stressful marital transitions as a risk factor for cognitive decline and potentially AD. While widowhood and divorce have not been explicitly identified as one of the major lifestyle risk factors for dementia, their experience may increase the frequency and intensity of a constellation of unhealthy behaviors identified by the Lancet Commission.^42^ These include, but are not limited to, excessive smoking and drinking as deleterious coping strategies for these stressful life events, increased symptoms of depression, social withdrawal and isolation after spousal loss, and increased risk of diabetes and cardiovascular illnesses.^6^ Moreover, our findings related to Aβ pathology imply that dementia risk cannot be wholly ascribed to these other risk factors. Marital dissolution may warrant consideration as a life course dementia risk factor in its own right.

Statistics demonstrate an increasing rate of divorce,^43^ and global projections suggest increases in advanced aging,^44^ likely indicating a higher future prevalence of widowhood. Thus, offering these individuals resources to cope with the stress of marital transitions may be justified as a public health priority to promote cognitive health and well‐being. This might include interpersonal psychotherapy to manage bereavement and loss.^45^ Findings from the current work also suggest that this group may warrant consideration as a priority for access to emerging anti‐amyloid disease‐modifying therapies for AD.^46^

While no mediation effects were observed when stratifying groups by sex, secondary results indicated that among individuals who had their marriage dissolved, women had higher EM performance than men. This finding is consistent with studies which found that divorce was more strongly associated with a higher risk of dementia for men than for women.^6^ Based on existing data,^47^ future studies may be warranted to more comprehensively examine whether the loss of a spouse may hurt men's health and well‐being, including cognitive health, more than women's. Additionally, this may shed light on whether men, more than women, are likely to benefit from interventions designed to counteract the adverse psychological effects of losing a spouse. However, there was also a noticeable underrepresentation of men in the marital dissolution group, which raises the possibility of ascertainment bias^48^ during data collection in ADNI that may have influenced this finding. We aimed to address this through sex‐stratified analyses.

Despite its novelty, the current study has several limitations that need to be addressed. First, we used a cross‐sectional study design, so temporal influences of marital dissolution could not be assessed. Moreover, as the ADNI study excluded subjects with major depressive disorder or significant depressive symptomatology (Geriatric Depression Scale [GDS] Score > 6), we did not include any measures of depression. A lack of such psychological measures makes it difficult to directly test our stress hypothesis or loss of social support in explaining our results. Our sample was also limited in terms of race, with > 90% consisting of White participants. These results require validation in cohorts consisting of a range of ethnicities including, but not limited to, Black and South Asian individuals who may be at elevated risk of dementia and have a younger age of dementia onset^49^ and individuals from different cultural backgrounds who may have varying familial support structures. Moreover, while we combined widowed and divorced individuals into one group due to the hypothesized shared stress and to maximize statistical power, there may be key differences among these groups. These include older ages for widows/widowers, additional psychological burden in the widowhood group due to bereavement, and the unique financial impacts of divorce. While rates of divorce in older age or “gray divorce” have accelerated in recent decades,^50^ it is likely that many participants in our study experienced divorce earlier in their life course and may have recovered from its effects. Conversely, for widowed participants, spousal loss may have occurred closer to the time of study entry. Similar to our approach, previous research has combined participants who were divorced and widowed into one category due to concerns of reduced power when examining outcomes associated with the groups individually.^2^

Data on marital status were also only collected at one time point during study entry. Although the number of average months between baseline and the initial amyloid PET scan visits was low, it is possible that some participants who were initially married at baseline had lost their partners by the time of their scan. A salient limitation of this study was that data on the duration of time after widowhood or divorce were not available in ADNI, which may be important in understanding the effects of stress associated with more acute events compared to more distant ones. Additionally, data were not available that may have helped clarify the nature of our findings. This includes the quality of marriage before dissolution, whether the married group consisted of individuals in remarriage or their first marriage, and the marital biographies of respondents to examine life course effects and exits from the divorced or widowed group into remarriage. One possibility is that individuals with greater socioeconomic or psychosocial resources may have been more likely to remarry after spousal loss, while those remaining in the divorced/widowed group may have had additional risk factors that contributed to poorer memory performance. Future research should consider these limitations and aim to validate these findings in highly powered and diverse distinct marital status groups.

## AUTHOR CONTRIBUTIONS

Avinash Chandra and Charles R. Marshall conceptualized the study. Avinash Chandra obtained access, and cleaned and curated the data from ADNI. Avinash Chandra and Sheena Waters conducted the statistical analysis. Avinash Chandra drafted the original manuscript and generated all tables and figures. All authors provided a critical review of and edits to the manuscript before submission.

## CONFLICT OF INTEREST STATEMENT

S.W. has received funding from UKRI Innovate UK. Professor C.R.M. has received research grant funding from NIHR, Innovate UK, Michael J. Fox Foundation, Alzheimer's Research UK, and Tom and Sheila Springer Charity. A.C., R.A., P.P., and L.J.S. declare no conflicting interests related to this work. Author disclosures are available in the supporting information.

## CONSENT STATEMENT

Informed consent was obtained from all human subjects within ADNI for whom the data were used in this study.

## Supporting information



Supporting Information

Supporting Information
