# Supplementary material for: Marital dissolution and cognition: The mediating effect of Aβ neuropathology
Source: Alzheimers Dement (Amst). 2024 Oct 29;16(4):e70032. doi: 10.1002/dad2.70032 (PMC11520438; doi:10.1002/dad2.70032)
Supplement: Supplementary file 1 — Supporting Information [file DAD2-16-e70032-s002.docx]

**Supplementary Material**

**Figure S1.** Frequency distribution for amyloid data. This histogram indicates the distribution of Aβ Centiloid values used in combination with the Shapiro-Wilk tests to identify non-normal distributions.


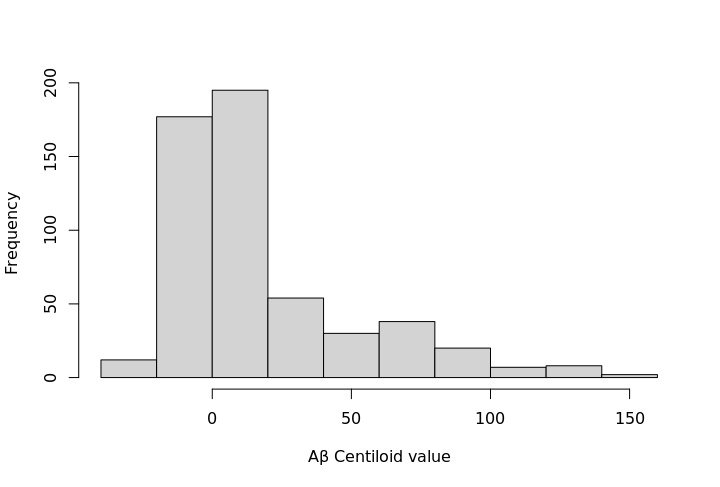


**Figure S2.** Frequency distribution for executive functioning performance data. This histogram indicates the distribution of executive functioning scores used in combination with the Shapiro-Wilk tests to evaluate normality of distributions.


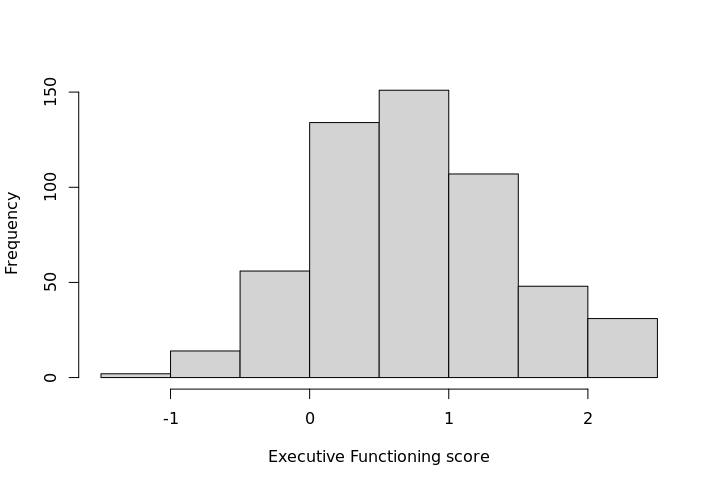


**Figure S3.** Frequency distribution for episodic memory performance data. This histogram indicates the distribution of episodic scores used in combination with the Shapiro-Wilk tests to evaluate normality of distributions.


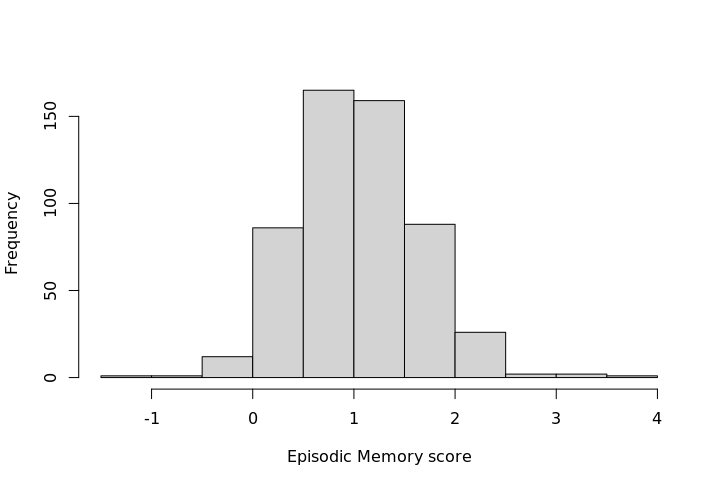


**Figure S4.** QQ plot for amyloid data. This QQ plot indicates the presence of an extreme outlier for Aβ Centiloid values, which was subsequently removed.


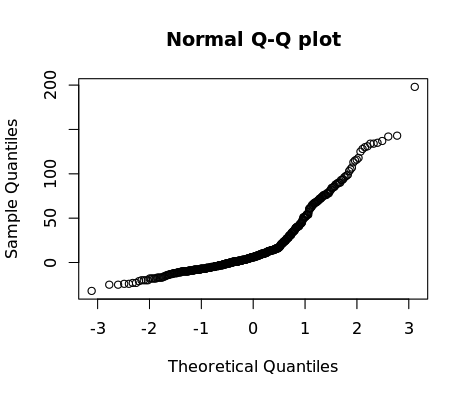


**Table S1.** Results from normality testing. This table presents results from Shapiro-Wilk tests of normality.

| **Variable** | **Shapiro-Wilk Value (W)** | ***P* Value** |
| --- | --- | --- |
| Aβ Centiloid value | 0.82 | <2.2e-16^***^ |
| EF Score | 0.99 | 0.0008^***^ |
| EM Score | 0.99 | 0.005^**^ |

^***^Significant at *P*<0.001

^**^Significant at *P*<0.01

Significant value indicates violation of normality assumption

Abbreviations: EF, executive functioning; EM, episodic memory.

**Table S2.** Secondary models with interaction terms. This table presents results from analyses examining influence interaction terms that include marital dissolution on (1) Aβ and (2) episodic memory performance.

| **Regression Model** | **Interaction Term** | **β** | **95% Confidence Interval** | **t-value** | ***P* Value** |
| --- | --- | --- | --- | --- | --- |
| Model 1: Aβ | Marriage Dissolution * Age | –0.02 | –0.08 to 0.04 | –0.56 | 0.574 |
| Model 2: Aβ | Marriage Dissolution * Sex | 0.19 | –0.79 to 1.18 | 0.39 | 0.700 |
| Model 3: Aβ | Marriage Dissolution * Education | –0.04 | –0.22 to 0.13 | –0.48 | 0.628 |
| Model 4: Aβ | Marriage Dissolution * *APOE* ε4 (heterozygous) | 0.66 | –0.35 to 1.68 | 1.28 | 0.201 |
| Model 4: Aβ | Marriage Dissolution * *APOE* ε4 (homozygous) | 0.96 | –2.47 to 4.40 | 0.55 | 0.581 |
| Model 5: EM score | Marriage Dissolution * Age | –0.004 | –0.01 to 0.004 | –1.07 | 0.285 |
| Model 6: EM score | Marriage Dissolution * Sex | 0.20 | 0.07 to 0.33 | 2.96 | 0.003^**^ |
| Model 7: EM score | Marriage Dissolution * Education | 0.003 | –0.02 to 0.03 | 0.28 | 0.780 |
| Model 8: EM score | Marriage Dissolution * Aβ | 0.003 | –0.02 to 0.03 | 0.26 | 0.794 |

^**^Significant at *P*<0.01

Abbreviations: EM, episodic memory

**Sensitivity Analysis Results:**

When stratifying the sample by sex, no significant interaction effects were found between Aβ and marital dissolution for males (β= 0.03; 95% CI: –0.03 to 0.08; *P*= 0.321) or females (β= –0.02; 95% CI: –0.05 to 0.01; *P*=0.154) on memory scores. Moreover, no significant mediation effects were observed when stratifying by sex. Specifically, for males, no significant association between marriage dissolution and Aβ pathology (Criteria 2; β= 0.41; 95% CI: –0.47 to 1.29; *P*= 0.358) or between Aβ pathology and memory performance when controlling for marital dissolution (Criteria 3; β= –0.02; 95% CI: –0.04 to 0.003; *P*= 0.092) were found. For females, no significant associations were found between marriage dissolution and memory performance (Criteria 1; β= –0.04; 95% CI: –0.10 to 0.02; *P*= 0.163) or between Aβ pathology and memory performance when controlling for marital dissolution (Criteria 3; β= –0.01; 95% CI: –0.02 to 0.003; *P*= 0.143).

In fully adjusted models (accounting for age squared), Baron and Kenney criteria for partial mediation was still met. Specifically, significant associations were observed between marriage dissolution and memory performance (Criteria 1; β= –0.09; 95% CI: –0.15 to –0.03; P= 0.003), marriage dissolution and Aβ pathology (Criteria 2; β= 0.59; 95% CI: 0.14 to 1.05 *P*= 0.011), and Aβ levels and memory scores when including marital dissolution as a covariate (Criteria 3; β= –0.01; 95% CI: –0.02 to –0.002; *P*= 0.021). When including Aβ pathology in the regression equation, the relationship between marriage dissolution and EM scores becomes weaker in terms of effect size and significance value (Criteria 4; Table S3). Results from non-parametric bootstrapping confirmed that both direct and Aβ-mediated effects for the association between marriage dissolution and memory performance were still present when including age squared into models (Table S4).

When examining only widowed participants, no significant mediation effects were observed. Specifically, no significant association between widowhood and Aβ pathology (Criteria 2; β= 0.51; 95% CI: –0.13 to 1.15; *P*= 0.120) was found. In addition, no significant interaction effect (β= 0.01; 95% CI: –0.02 to 0.05; *P*= 0.415) was found between Aβ and widowhood on memory scores. We also examined divorced participants in isolation and found no mediation effects. There was no significant association between divorce and memory performance (Criteria 1; β= –0.06; 95% CI: –0.13 to 0.01; *P*= 0.098). Additionally, there were no separate associations between widowhood (β= –0.05; 95% CI: –0.20 to 0.10; *P*= 0.516) or divorce (β= –0.06; 95% CI: –0.19 to 0.07; *P*= 0.374) and executive functioning performance.

**Table S3.** Mediation results using Baron and Kenney criteria including age squared. This table presents partial mediation results with and without Aβ as a covariate with age squared included in models.

| **Regression Model** | **β^a^** | **95% Confidence Interval^a^** | **t-value** | ***P* Value** |
| --- | --- | --- | --- | --- |
| (1) EM score ~ Marriage Dissolution | –0.093 | –0.154 to –0.032 | –2.99 | 0.003^**^ |
| (2) EM score ~ Marriage Dissolution + Aβ | –0.087 | –0.148 to –0.026 | –2.79 | 0.006^**^ |

^a^Rounded to 3 decimal places to demonstrate differences in effect size

^**^Significant at *P*<0.01

Abbreviations: EM, episodic memory

**Table S4.** Mediation results using bootstrapping including age squared. This table presents results from mediation results using nonparametric bootstrap confidence intervals with the percentile method with age squared included in models.

| **Outcome** | **Estimate^a^** | **95% Confidence Interval^a^** | ***P* Value** |
| --- | --- | --- | --- |
| Average causal mediation effect | –0.008 | –0.018 to –0.0007 | 0.023^*^ |
| Average direct effect | –0.087 | –0.166 to –0.012 | 0.018^*^ |
| Total effect | –0.094 | –0.171 to –0.021 | 0.009^**^ |
| Proportion Mediated | 0.080 | 0.005 to 0.403 | 0.031^*^ |

^a^Rounded to 3 decimal places to highlight precise effect size

^*^Significant at *P*<0.05

^**^Significant at *P*<0.01

Abbreviations: EM, episodic memory
